# Peer Review of “Improving Tuberculosis Detection in Chest X-Ray Images Through Transfer Learning and Deep Learning: Comparative Study of Convolutional Neural Network Architectures”

**DOI:** 10.2196/77174

**Published:** 2025-07-01

**Authors:** Natthapong Nanthasamroeng

**Affiliations:** 1 Ubon Ratchathani Rajabhat University, Ubon Ratchathani, Thailand

**Keywords:** tuberculosis detection, tuberculosis, TB, chest x-ray classification, diagnostic imaging, radiology, medical imaging, convolutional neural networks, data augmentation, deep learning, early warning, early detection, comparative study

*This is a peer-review report for “Improving Tuberculosis Detection in Chest X-Ray Images Through Transfer Learning and Deep Learning: Comparative Study of Convolutional Neural Network Architectures.”*

## Round 1 Review

### General Comments

The manuscript [1] presents a study that evaluates the performance of various convolutional neural network architectures—namely, VGG16, VGG19, ResNet50, ResNet101, ResNet152, and Inception-ResNet-V2—in classifying chest x-ray images to detect tuberculosis (TB). The authors compare the models’ classification accuracy, precision, recall, *F*_1_-score, and area under the receiver operating characteristic curve, concluding that VGG16 outperforms the others with high accuracy and efficiency. They also assess the impact of data augmentation, finding it does not improve model performance due to sufficient diversity in the original dataset.

### Specific Comments

1. The dataset includes a large imbalance between TB-positive and TB-negative images (700 vs 3500). Explain how this imbalance was addressed beyond augmentation or whether balancing techniques like oversampling were considered.
2. While each architecture’s parameters are listed, there is no in-depth discussion on why these specific parameters (eg, dropout rates, learning rates) were selected.
3. The conclusion that data augmentation did not improve performance lacks specific references to possible reasons.
4. While computational time for each model is reported, further analysis of the practical implications, such as cost-effectiveness for clinical settings, is missing.
5. The manuscript mentions transfer learning with pretrained ImageNet weights, but there is limited information on why this was the chosen approach versus training from scratch.
6. Throughout the Results section, adding comparative charts or visual aids for each model’s performance across metrics like accuracy, precision, and area under the receiver operating characteristic curve would improve readability.
7. The Conclusion could benefit from a clearer statement on how these findings advance the field of TB detection in medical imaging.

## Abbreviations

TB: tuberculosis
